# Salient object detection dataset with adversarial attacks for genetic programming and neural networks

**DOI:** 10.1016/j.dib.2024.111043

**Published:** 2024-11-04

**Authors:** Matthieu Olague, Gustavo Olague, Roberto Pineda, Gerardo Ibarra-Vazquez

**Affiliations:** a IBM Technology Campus Guadalajara, El Salto, 45680, Mexico; b Department of Computer Science, CICESE, Ensenada, 22860, México; c Department of Data Science, ITESM, Monterrey, 64849, México

**Keywords:** Symbolic learning, Deep learning, Visual attention, Adversarial robustness, Adversarial examples

## Abstract

Machine learning is central to mainstream technology and outperforms classical approaches to handcrafted feature design. Aside from its learning process for artificial feature extraction, it has an end-to-end paradigm from input to output, reaching outstandingly accurate results. However, security concerns about its robustness to malicious and imperceptible perturbations have drawn attention since humans or machines can change the predictions of programs entirely. Salient object detection is a research area where deep convolutional neural networks have proven effective but whose trustworthiness represents a significant issue requiring analysis and solutions to hackers' attacks. This dataset is an image repository containing five different image databases to evaluate adversarial robustness by introducing 12 adversarial examples, each leveraging a known adversarial attack or noise perturbation. The dataset comprises 56,387 digital images, resulting from applying adversarial examples on subsets of four standard databases (i.e., FT, PASCAL-S, ImgSal, DUTS) and a fifth database (SNPL) portraying a real-world visual attention problem of a shorebird called the snowy plover. We include original and rescaled images from the five databases used with the adversarial examples as part of this dataset for easy access and distribution.

Specifications Table

**Table:** 

Subject	Computer Vision and Pattern Recognition
Specific subject area	Salient object detection, Deep convolutional neural networks, Symbolic learning, Adversarial robustness, Visual attention
Type of data	Image
Data collection	This repository contains five different image databases that help evaluate adversarial robustness. Four databases show common everyday objects (i.e., FT, ImgSal, PASCAL-S, DUTS). The fifth database, named the SNPL database, shows 250 images of snowy plovers. Considering different weather, exposure, and range conditions, we took photos using a Nikon DSLR camera and a 200-500mm f/5.6 super-telephoto lens. Twelve adversarial examples (AEs) were generated for the validation set of each image database using the Python programming language, version 3.8.3, and the machine learning framework PyTorch, version 1.6.0.
Data source location	The FT database was recovered from [1]. The ImgSal database was recovered from [2]. The PASCAL-S database was recovered from [3]. The DUTS database was recovered from [4]. We collect the SNPL database from the snowy plover's natural habitat, consisting of sandy beaches and mudflats on the Pacific coast of Baja California, Mexico.
Data accessibility	Repository name: Adversarial Attacks for Salient Object Detection Data identification number: 10.17632/98zpfhg7vn.1 Direct URL to data: https://data.mendeley.com/datasets/98zpfhg7vn/1 Instructions for accessing these data: The dataset is available at Mendeley Data [5].
Related research article	G. Olague et al., ``Adversarial Attacks Assessment of Salient Object Detection via Symbolic Learning,'' in IEEE Transactions on Emerging Topics in Computing, vol. 11, no. 4, pp. 1018-1030, Oct.-Dec. 2023, doi: 10.1109/TETC.2023.3316549.

## 1 Value of the Data

• This dataset can allow researchers to validate the robustness of a symbolic salient object detection (SOD) algorithm against adversarial attacks (AAs) by conducting a deeper analysis and contrasting it with deep learning (DL) models, twelve AEs, and five different datasets.
• The dataset presented in this paper is a ready-to-use database that includes commonly used, manually collected, and artificially generated images that can help researchers working in SOD develop new algorithms which somebody can then integrate into various other applications.
• The SNPL dataset represents a challenging scenario to test different SOD models. Researchers could compare with the other included standard datasets (i.e., FT, PASCAL-S, IMGSAL, DUTS) to understand the limitations of academic datasets whose optimal size and exposure conditions prevent us from understanding the effect of AEs on visual attention.

## 2 Background

AAs are a relevant topic in the current study of DL models; initially, AAs have attracted attention to image classification models since researchers first discovered the phenomenon. Soon, the subject spread to diverse areas where researchers started to demonstrate to appear as a vulnerability in DL modeling. In visual attention, given a set of images I={I1,I2,...,In} and their corresponding proto-objects P={P1,P2,...,Pn}, the deep SOD model establishes a relationship between an image (Ii) and the proto-object (Pi). AAs denote the erroneous behavior of such models when the input image suffers a slight change in its pixels by adding a perturbation ρ to create the AE; Iρ=I+ρ. The AE is considered the intentionally modified input image that is recognized differently than the I.

The original research article [6] provides extensive evidence and insight on how AAs break DL models, rendering them vulnerable. This data article adds further value to the subject by allowing researchers to validate the robustness of a SOD algorithm against AAs (according to sub-symbolic methods, symbolic methods, and brain programming, which is a mixture of symbolic learning (genetic programming) with classical computer vision) and also by providing a dataset including commonly used, manually collected, and artificially generated images that researchers working in SOD can use as a testbed to develop new algorithms and integrate them into various other applications.

## 3 Data Description

The adversarial attacks for salient object detection dataset is an image repository built to evaluate the adversarial robustness of SOD algorithms through five different image databases and 12 AEs that leverage known AAs or noise perturbations. The repository contains a total of 56,387 digital images through the application of AEs to four standard databases (i.e., FT, PASCAL-S, ImgSal, DUTS), as well as a fifth database (SNPL) portraying a real-world visual attention problem of a shorebird called the snowy plover.

**Fig. 1:**
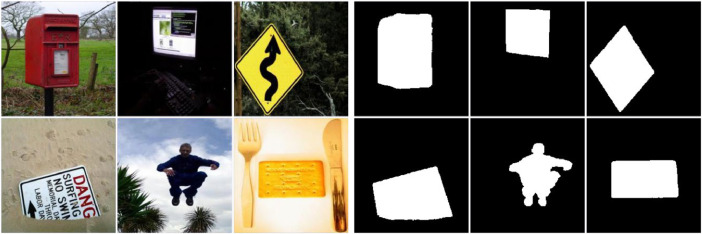
Some example images of the FT database with the corresponding ground truth.

**Fig. 2:**
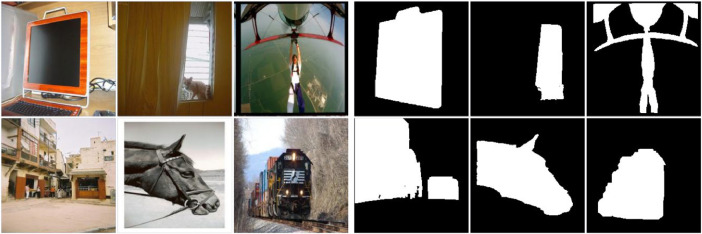
Some examples of PASCAL-S database images with the corresponding ground truth.

**Fig. 3:**
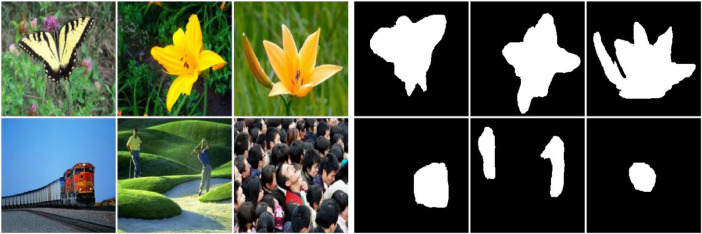
This collage shows a few example images of the IMGSAL database with the corresponding ground truth.

**Fig. 4:**
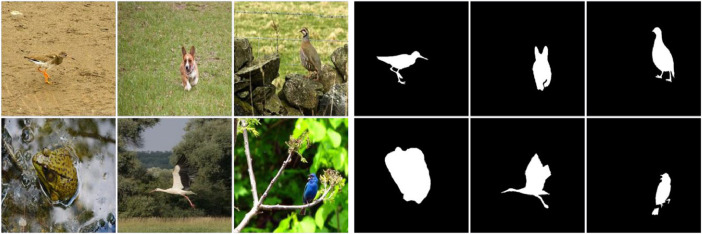
Some example images of the DUTS database with the corresponding ground truth.

The FT database, see Fig. 1, contains diverse portraits of animate and inanimate objects with images ranging from 324 × 216 to 400 × 300 pixels. We selected 700 random images from this dataset for training and 300 for testing. PASCAL-S consists of 850 images, from which we selected 595 images for training and 255 for testing (Fig. 2). PASCAL-S contains scenes of domestic animals, persons, and means of air and sea transport, with images ranging from 200 × 300 to 375 × 500 pixels. IMGSAL provides 235 images, from which we reserved 165 for training, and 70 were randomly chosen for testing (Fig. 3). The dataset includes wild animals, flora, and different objects and people with image sizes of 480 × 640 pixels. Regarding the last of the standard datasets, DUTS contains 10,553 training images and 5,019 test images portraying people, animals, insects, and objects in a wide variety of scenarios (Fig. 4). We selected 700 training images and 300 testing images at random from DUTS original sets, which have sizes ranging from 266 × 400 to 400 × 192 pixels.

**Fig. 5:**
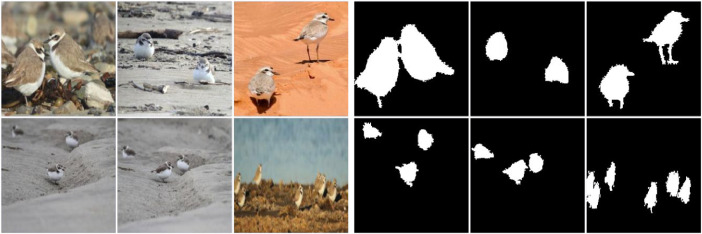
Some example images of the SNPL database with the corresponding ground truth.

The fifth database used in this work comprises 250 images of snowy plovers and is named the SNPL database. Fig. 5 shows pictures taken using a Nikon DSLR camera and a 200-500mm f/5.6 super-telephoto lens, considering different weather, exposure, and range conditions. The photographs represent the bird's natural habitat, consisting of sandy beaches and mudflats on the Pacific coast of Baja California, Mexico. Each image shows one or more snowy plovers, whose size on the scene varies according to the distance at which the photograph was taken while adjusting the focal length. Another essential feature of the SNPL dataset involves the background, where various distractors, such as plants, water bodies, and small objects (shells and debris), are present. Also, many images contain occlusions that partially cover the object of interest. Note that the animal's sandy beach habitat and the primary color of its feathers have similar color tones. Many of these images lack a high contrast between the background and the snowy plovers.

**Fig. 6:**
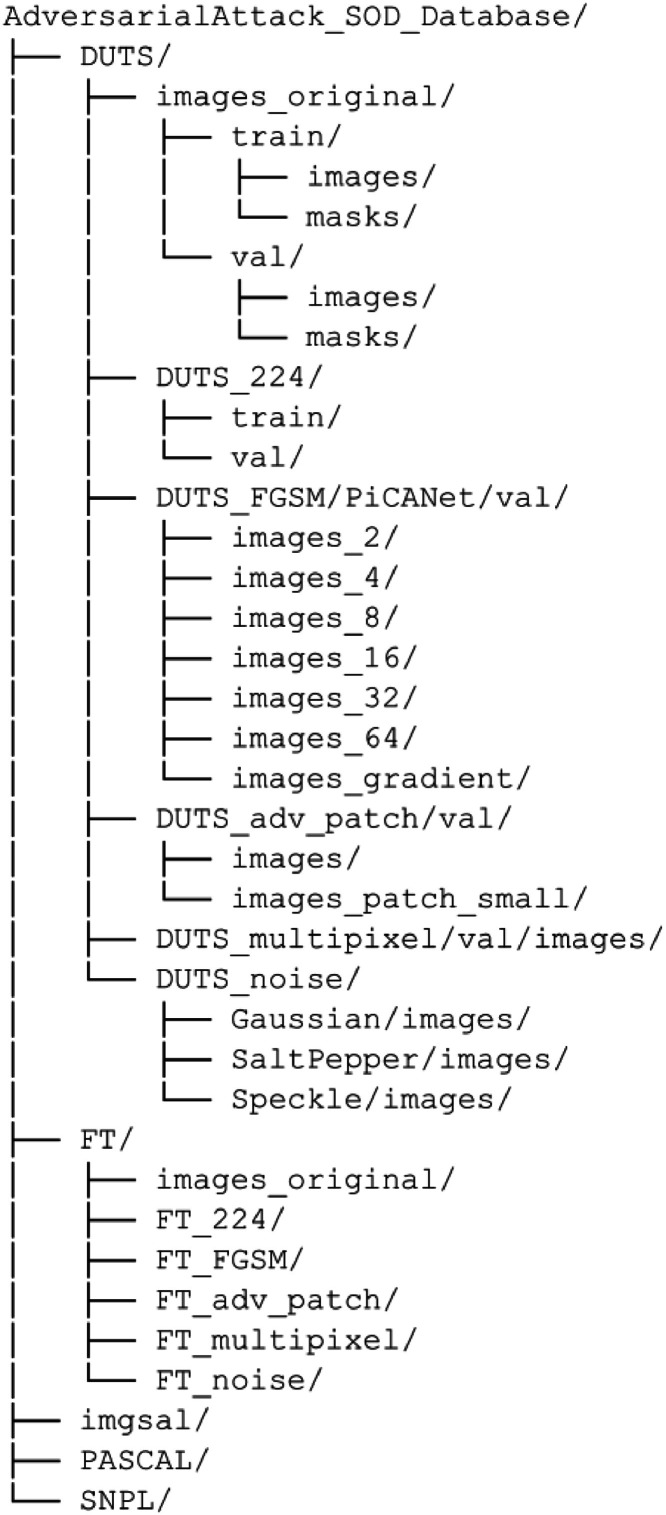
A folder structure diagram shows the pattern used to organize the dataset.

Each dataset is in its folder with all original images plus rescaled images of 224 × 224 pixels, whose size corresponds to most inputs required by DL models. We applied AAs to the testing set of each database. We stored it in four different subfolders, each alluding to the type of AA used (i.e., Adversarial Patch (AP), Fast Gradient Sign Method (FGSM), Multipixel Attack (MA), and Noise). The twelve AEs generated for each image in the dataset can be referred to as FGSM with epsilon value ε={2,4,8,16,32,64}, AP, AP-small, MA, Gaussian Noise, Salt & Pepper Noise, and Speckle Noise. Fig. 6 helps to visualize the folder structure of the AAs for the SOD dataset.

## 4 Experimental Design, Materials and Methods

### 4.1 Adversarial attacks

AAs involve manipulating or exploiting a machine-learning model using carefully crafted data. Next, we describe four different AAs used in the database construction. These were selected to represent the most common strategies present in literature for classifying perturbations.

On the one hand, AAs can be classified based on the model's available information. White box attacks possess complete access to the model's architecture, the parameter values, and the trained task. Conversely, black box attacks aim to change the original prediction without knowing the model, which we achieved through a testing procedure for optimizing the perturbation.

In addition, other methodologies extend the abovementioned strategies by specifying whether their goal is to trick a model into selecting a specific label (targeted attack) or to predict any label other than the original one (untargeted attack).

The dataset also seeks to test the transferability effect, a phenomenon observed when AEs built for a specific model and task still manage to affect other deep learning models not necessarily trained to solve the same problem.

Hence, the selected attacks are a white box untargeted (FGSM), a black box untargeted (MA), a white box targeted attack (AP), and additional randomly perturbed black box untargeted attacks (Noise-based). Since white box attacks used a particular model to generate the AEs, they also serve as a suitable means to test the transferability effect. The following subsections explain these perturbations in detail.

#### 4.1.1 Fast Gradient Sign Method (FGSM)

Goodfellow et al. first implemented FGSM by noting that linear behaviors of high-dimensional spaces easily generate AEs [7]. FGSM proposes to increase the loss of the detector by solving the following equation: ρ=εsign(ΔJ(θ,I,G)), where ΔJ() computes the gradient of the cost function around the current value of the model parameters θ for the image I, and the ground truth G. sign() denotes the sign function, which maximizes the magnitude of the loss, and ε is a small scalar value that restricts the norm $L_{\infty }$ of the perturbation.

#### 4.1.2 Multipixel Attack (MA)

The multipixel adversarial perturbation is a black box untargeted attack since it does not require network information [8]. Although a one-pixel attack is the basis, such an algorithm is unsuitable for real-world problems since it can only work for icon images. The multipixel version has the intention of testing attacks with bigger images. In summary, let the vector I=(I1,...,In) be an n-dimensional image, which is the input of the salient object detector f() that correctly predicts the object t from the image. The statistics of I associated with the object t is Z(f(I),f(I+e(I))). It builds an additive adversarial perturbation vector e(I)=(e1,...,en) according to I, the salient object detector, and the limitation of maximum modifications d, a small number that expresses the dimensions that are modified, while other dimensions of e(I) that are left are zeros. The goal is to find the optimal solution $e{\left(i\right)}^{*}$ that solves the following equation:

$$\min_{e{\left(i\right)}^{*}}Z\left(f\;\left(I\right),\;f\;\left(I\right)+\;e\left(I\right)\right)$$

$$s.t.\;{\left|\left|e\left(I\right)\right|\right|}_{0}\leq d$$

#### 4.1.3 Adversarial Patch (AP)

The AP is a white box attack that creates a particular form of perturbation, which builds a universal perturbation with a patch shape [9]. Even though this attack is for image classification tasks, its universality makes it perfect for analyzing the transferability effect that this perturbation provokes. The AP builds the perturbation, maximizing $ftarget(I\;+\;\hat{p}))$ to a specific class where it finds the optimal patch $\hat{p}$. Universal perturbations such as the AP pose a powerful, foolproof attack that relies on various transformations applied to the patch to fool the system. Moreover, some suggest printing the resulting example attacks to test it in real-world conditions. The algorithm trains the AP $\hat{p}$ using a set of images I, and a variant of the expectation over transformation framework to optimize the following equation:

$$\hat{p}=\underset{\hat{p}}{\text{argmax}}E_{I\in I.t\in T.l\in L}\left[log\;f\;\left(ytarget,\;A\left(p,\;I,\;l,\;t\right)\right)\right].$$

Where ytarget represents the target class in the image classification model and A(p,I,l,t) is a function that first applies the transformation t from a distribution over transformations T to the patch p. Next, it puts the transformed patch p at the location l from a distribution over locations L to the image I. The effectiveness of these patches resides in the expectation of the training images that increase success, regardless of the background.

#### 4.1.4 Noise-based attacks

Some people usually interpret AEs as strategically perturbed images that fool DL models. However, randomly perturbed images are also studied to verify the robustness of SOD models. We include three types of noise (Gaussian, Salt & Pepper, and Speckle) to study the behavior of such algorithms with randomly perturbed inputs. Gaussian noise adds a white noise with a probability density function of a normally distributed random variable with an expected value μ and variance σ. The general probability density function is as follows:

$$g\left(x\right)=\;\frac{1}{\sigma \sqrt{2\pi }}e^{\frac{-{\left(x-\mu \right)}^{2}}{2\sigma^{2}}}$$

Salt & Pepper noise with density (d) assigns each pixel (p) from the total pixels (tp) in an image, a random probability value (pv) from a standard uniform distribution between (0,1). Therefore, it applies to the cases listed below.

• p=0, for pv∈(0,d/2) limited to d×tp/2 pixels.
• p=maxvalue, for pv∈[d/2,d) limited to d×tp/2 pixels.
• p=p, for pv∈[d,1)

Speckle noise adds a multiplicative perturbation using the following equation J=I+m×I, where m is a uniformly distributed random noise with μ=0 and σ=0.05.

### 4.2 Generation of AEs

This work tests the robustness of SOD algorithms to AAs by creating a validation set of each database using the Python programming language, version 3.8.3, and the PyTorch machine learning framework, version 1.6.0 [10].

**Fig. 7:**
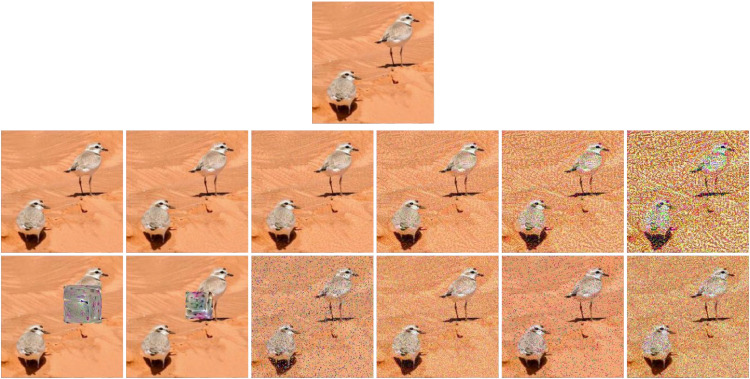
illustrates some perturbations over an image of the SNPL database. On top is the original image, while in the second row, we observe the image with different levels of perturbation using FGSM; the third row provides the resulting AEs using two versions of the AP, the MA, and the Gaussian, Salt & Pepper, and Speckle noises respectively.

The article considers six sets of AEs generated with FGSM, coupling this attack with the training stage of PICANet [11]. Each set used a value of ε={2,4,8,16,32,64}, allowing resulting images with disturbances of different intensities. An ε value of 2 corresponds to the lowest perturbation, and 64 provides the highest variation to the original image. In the second row of Fig. 7, there is an example of how one image of snowy plovers becomes increasingly altered with higher values of ε.

We generated two sets of AEs for the AP using ResNet-50 [12]. The first set's images have patches covering an area of 70 × 70 pixels. In comparison, patches in the second set are smaller, with dimensions of 50 × 50 pixels (Fig. 7). The objective behind having patches of different sizes is to enable assessments of whether the performance of the detection algorithm is dependent on the patch size, or if in the end, the disturbance is the main contributing factor to erroneous behavior. We placed the patch randomly, regardless of location, even if it covered the object of interest wholly or partially.

In addition, we generated an AE using the MA method, which used a disturbance value d=10,000, specifying the number of modified pixels. Using this value resulted in many altered pixels, which remain noticeable while allowing objects in the image to stay perceptible. Since MA is a black box attack, there is no need to know the internal parameters used for the machine learning model.

Finally, for the noise-based AEs, one set was generated for each database using Gaussian noise. With a value of σ = 30, this resulted in a grain with sufficient magnitude to make the attack visible without obscuring the objects in the scene. We also generated sets using Salt & Pepper noise, and since color images constitute the databases, the noise appears as colored dots. The effect results from the Salt & Pepper noise taking values at each color band while considering the maximum and minimum pixels and their distribution across the image. Another noise-based AE uses a Speckle noise, which simulates the original physical phenomenon by multiplying uniformly distributed random values along image dimensions by a variance of 0.3. It's worth noting that none of the noise attacks required knowledge of the internal parameters of any learning approach, adhering to the black box classification of attacks. Fig. 7 shows these AEs applied to an image from the SNPL dataset.

## Limitations

The included academic databases (i.e., FT, PASCAL-S, IMGSAL, DUTS) have optimal size and exposure conditions that do not represent a real-world problem missing the characteristics of being in their natural and undisturbed environment, contrary to the SNPL database. Future work can include more real-world databases to address this limitation, exemplifying other problems linked to Computer Vision and Image Processing, for instance, by including images in the fields of medicine or surveillance or directly working in a technique like 3D reconstruction or object tracking.

Furthermore, while the chosen AEs represent the most common perturbation classification strategies, we suggest adding more types of AAs in future work. Nevertheless, the included attacks are sufficient to demonstrate the robustness of a studied SOD algorithm.

## Ethics Statement

All authors of this article have read and followed the ethical requirements for publication in Data in Brief and confirm that the current work does not involve human subjects, animal experiments, or any data collected from social media platforms.

## Credit Author Statement

**Matthieu Olague:** Software, Validation, Data Curation, Writing – Original Draft, Writing – Review & Editing, Visualization. **Gustavo Olague:** Conceptualization, Methodology, Validation, Writing – Review & Editing, Supervision, Project administration, Resources, Funding acquisition. **Roberto Pineda:** Software, Methodology, Investigation, Data Curation. **Gerardo Ibarra Vazquez:** Conceptualization, Methodology, Investigation.

## Data Availability

Mendeley DataAdversarial Attacks for Salient Object Detection (Original data).
